# Supplementary material for: Haematological dynamics following treatment of visceral leishmaniasis: a protocol for systematic review and individual participant data (IPD) meta-analysis
Source: BMJ Open. 2023 Dec 14;13(12):e074841. doi: 10.1136/bmjopen-2023-074841 (PMC10729213; doi:10.1136/bmjopen-2023-074841)
Supplement: Author's manuscript [file bmjopen-2023-074841.draft_revisions.pdf]

BMJ Open

BMJ Open is committed to open peer review. As part of this commitment we make the peer review history of every article we publish publicly available.

When an article is published we post the peer reviewers' comments and the authors' responses online. We also post the versions of the paper that were used during peer review. These are the versions that the peer review comments apply to.

The versions of the paper that follow are the versions that were submitted during the peer review process. They are not the versions of record or the final published versions. They should not be cited or distributed as the published version of this manuscript.

BMJ Open is an open access journal and the full, final, typeset and author-corrected version of record of the manuscript is available on our site with no access controls, subscription charges or pay-per-view fees (http://bmjopen.bmj.com).

If you have any questions on BMJ Open's open peer review process please email info.bmjopen@bmj.com

BMJ Open

Haematological dynamics following treatment of visceral leishmaniasis: a protocol for systematic review and individual participant data (IPD) meta-analysis

Journal:	*BMJ Open*
Manuscript ID	bmjopen-2023-074841
Article Type:	Protocol
Date Submitted by the Author:	18-Apr-2023

Complete List of Authors:	Munir, Abdalla; Infectious Diseases Data Observatory (IDDO); University of Oxford, Centre for Tropical Medicine and Global Health, Nuffield Department of Clinical Medicine<br>Dahal, Prabin; University of Oxford, Centre for Tropical Medicine and Global Health, Nuffield Department of Clinical Medicine<br>Kumar, Rishikesh; Rajendra Memorial Research Institute of Medical Sciences (RMRIMS)<br>Singh-Phulgenda, Sauman; Infectious Diseases Data Observatory (IDDO); University of Oxford, Centre for Tropical Medicine and Global Health, Nuffield Department of Clinical Medicine<br>Siddiqui, Niyamat; Rajendra Memorial Research Institute of Medical Sciences (RMRIMS)<br>Naylor, Caitlin; Infectious Diseases Data Observatory (IDDO); University of Oxford, Centre for Tropical Medicine and Global Health, Nuffield Department of Clinical Medicine<br>Wilson, James; Infectious Diseases Data Observatory (IDDO); University of Oxford, Centre for Tropical Medicine and Global Health, Nuffield Department of Clinical Medicine<br>Buck, Gemma; Infectious Diseases Data Observatory (IDDO); University of Oxford, Centre for Tropical Medicine and Global Health, Nuffield Department of Clinical Medicine<br>Rahi, Manju; Indian Council of Medical Research (ICMR)<br>Alves, Fabiana; Drugs for Neglected Disease Initiative<br>Malaviya, Paritosh; Banaras Hindu University, Infectious Disease Research Laboratory, Department of Medicine, Institute of Medical Sciences<br>Sundar, Shyam; Banaras Hindu University, Infectious Disease Research Laboratory, Department of Medicine, Institute of Medical Sciences<br>Ritmeijer, Koert; Médecins Sans Frontières<br>Stepniewska, Kasia; Infectious Diseases Data Observatory (IDDO); University of Oxford, Centre for Tropical Medicine and Global Health, Nuffield Department of Clinical Medicine<br>Pandey, Krishna; Rajendra Memorial Research Institute of Medical Sciences (RMRIMS)<br>Guérin, Philippe J.; Infectious Diseases Data Observatory (IDDO); University of Oxford, Centre for Tropical Medicine and Global Health, Nuffield Department of Clinical Medicine<br>Musa, Ahmed; University of Khartoum, Institute of Endemic Diseases

Keywords:	Anaemia < HAEMATOLOGY, EPIDEMIOLOGY, INFECTIOUS DISEASES, Tropical medicine < INFECTIOUS DISEASES

SCHOLARONE™
Manuscripts

Haematological dynamics following treatment of visceral leishmaniasis: a protocol for systematic review and individual participant data (IPD) meta-analysis

Abdalla Munir[1,2,3], Prabin Dahal[1,2§], Rishikesh Kumar[4], Sauman Singh-Phulgenda[1,2], Niyamat Ali Siddiqui[4], Caitlin Naylor[1,2], James Wilson[1,2], Gemma Buck[1,2], Manju Rahi[5], Fabiana Alves[6], Paritosh Malaviya[7,] Shyam Sundar[7], Koert Ritmeijer[8], Kasia Stepniewska[1,2], Krishna Pandey[4], Phillipe J Guerin[1,2§], Ahmed Musa[3§]

[1]Infectious Diseases Data Observatory (IDDO), Oxford, UK

[2]Centre for Tropical Medicine and Global Health, Nuffield Department of Clinical Medicine, University of Oxford, Oxford, UK

[3] Institute of Endemic Diseases, University of Khartoum, Khartoum, Sudan

[4]Rajendra Memorial Research Institute of Medical Sciences (RMRIMS), Patna, India

[5]Indian Council of Medical Research (ICMR), New Delhi, India

[6]Drugs for Neglected Diseases initiative, Geneva, Switzerland

[7]Infectious Disease Research Laboratory, Department of Medicine, Institute of Medical Sciences, Banaras Hindu University, Varanasi, India

[8]Médecins Sans Frontières, Amsterdam, Netherlands

§Correspondence: *prabin.dahal@iddo.org*, *musaam2003@yahoo.co.uk*

For peer review only - http://bmjopen.bmj.com/site/about/guidelines.xhtml

Authors' email:

AM abdalla.munir@iddo.org

PD prabin.dahal@iddo.org

RK rishikeshkumar@live.in

SSP sauman.singh@iddo.org

NAS niyamatalisiddiqui@yahoo.com

CN caitlin.naylor@iddo.org

JW james.wilson@iddo.org

GB gemma.buck@iddo.org

MR drmanjurahi@gmail.com

PM paritosh_malaviya@yahoo.com

FA falves@dndi.org

SS drshyamsundar@hotmail.com

KR koert.ritmeijer@amsterdam.msf.org

KS kasia.stepniewska@iddo.org

PJG philippe.guerin@iddo.org

KP drkrishnapandey@yahoo.com

For peer review only

For peer review only - http://bmjopen.bmj.com/site/about/guidelines.xhtml

Abstract

Introduction: Visceral leishmaniasis (VL) is a parasitic disease with an estimated 50,000 to 90,000 new cases occurring annually. Anaemia is the most common haematological manifestation of visceral leishmaniasis (VL). However, the evolution of different haematological characteristics following treatment remains poorly understood. An individual participant data meta-analysis (IPD-MA) is planned to characterise the haematological dynamics in VL patients.

Methods and analysis: The Infectious Diseases Data Observatory (IDDO) VL data platform is a global repository of IPD from therapeutic studies identified through a systematic search of published literature (PROSPERO registration: CRD42021284622). The platform currently holds datasets from clinical trials standardised to a common data format. Contacting authors and principal investigators of the studies indexed in the IDDO VL data platform meeting the eligibility criteria for inclusion in this IPD-MA were invited to be part of the collaborative IPD-MA. Mixed effects multivariable regression models will be constructed to identify determinants of haematological parameters by taking clustering within study-sites into account.

Ethics and dissemination: The results of this analysis will be disseminated at conferences, IDDO website and any peer-reviewed publication arising will be made open source. Findings of this research will be critically important for the control programmes at regional/global levels, policy makers and groups developing new VL treatment.

For peer review only - http://bmjopen.bmj.com/site/about/guidelines.xhtml

For peer review only

PROSPERO registration: The systematic review component of the IPD-MA has the following PROSPERO registration: CRD42021284622.

Keywords

Visceral Leishmaniasis; Kala-azar; Statistical Analysis Plan, meta-analysis, individual participant data; anaemia, haemoglobin; platelets, white blood cells, red blood cells

Word count: 2841 (main text); abstract (220)

For peer review only

For peer review only - http://bmjopen.bmj.com/site/about/guidelines.xhtml

Strengths and limitations of this study

- Visceral Leishmaniasis clinical trials are relatively small, which limits the ability to identify predictors associated with it any clinical outcomes in any single study. An individual participant data (IPD-MA) will increase the statistical power to detect the predictors and moderators of associated with evolution of haemoglobin.

- The identification of studies eligible for inclusion in the IPD-MA has been made through a comprehensive literature search of all published studies since 1980 with pre-defined inclusion-exclusion criteria.

- This IPD-MA will utilise the VL repository of individual participant data hosted by Infectious Diseases Data Observatory (IDDO VL data platform). A major strength of this study is that data on the IDDO VL data platform is harmonised to a common standard based on an extensive consultation with the VL research community.

- Retrieval of data from trials published prior to 2000 can be a major challenge.

For peer review only

For peer review only - http://bmjopen.bmj.com/site/about/guidelines.xhtml

Introduction

Anaemia is the most common haematological manifestation of Visceral Leishmaniasis (VL)[1]. In patients with VL, anaemia can arise due to one or more associated factors: sequestration and haemolysis of red blood cells in the spleen associated with hypersplenism, bone marrow suppression caused by nutritional deficiencies such as iron, vitamin B12 and folate deficiencies, or due to clotting dysfunction leading to blood loss [1–5]. At the time of clinical diagnosis, haemoglobin levels are often around 7–10 g/dL but can be as low as 4 g/dL [1]. The severity of anaemia depends on the duration of clinical illness and can be exacerbated by comorbidities and iron deficiency [6,7].

After treatment with an antileishmanial drug, haematological improvement generally occurs within a few weeks with significant recovery expected within 4-6 weeks [3]. The trajectory of haematological recovery may be affected by patients' age or initial parasite load [8], but the influence of drug regimen and other patient characteristics remains poorly understood. To characterise the haematological changes in VL after treatment, and understand the role of host, parasite and drug related characteristics, we propose to undertake an individual participant data meta-analysis (IPD-MA) of therapeutic studies.

Objectives

The objectives of this IPD-MA are:

1. To identify the determinants of haemoglobin concentration at enrolment, at initial cure assessment and at definitive cure assessment
2. To characterise the haemoglobin dynamics following treatment
3. To identify the determinants of anaemia or severe anaemia at enrolment, at initial cure assessment and at definitive cure assessment

For peer review only - http://bmjopen.bmj.com/site/about/guidelines.xhtml

4. To describe white blood cells and platelets dynamics following treatment (data permitting)
5. To identify predictors of blood transfusion during treatment or follow-up (if available)

Methods and analysis: Patients, interventions and outcomes

Elements of the research aim (PICOT)

Population: Any patient enrolled in an interventional study with a confirmed or suspected diagnosis of VL defined by serological and/or parasitological testing.

Interventions: Any antileishmanial therapy

Comparator: Not restricted by the use of a comparator drug

Outcome: At least one of the following outcomes reported: anaemia (or haemoglobin measurements) at enrolment, anaemia and other haematological measurements at any post-baseline time-points

Time: Studies published on or after 1980

Criteria for study eligibility

Studies in the IDDO VL data platform [9] will be deemed eligible for the purpose of this analysis if they meet the following criteria:

- Prospective clinical efficacy studies on patients with confirmed or suspected VL either using microscopy/serology/molecular method (i.e. clinical diagnosis followed by a confirmatory method)

For peer review only - http://bmjopen.bmj.com/site/about/guidelines.xhtml

For peer review only

- Information is available on treatment regimen including drug, dose, and duration
- Data on anaemia (or either haemoglobin or haematocrit measurements) measured at enrolment

Desirable criteria

- Methodology used for haematological quantification, e.g. name of devices
- Information regarding if blood transfusion were given before or after treatment initiation
- Information of co-administration of iron supplement

Criteria for participant eligibility

The following minimum information are required for participants from each of the identified studies for inclusion in the IPD-MA analysis:

- Details of antileishmanial treatment(s) administered
- Baseline information on age and gender
- Outcome is recorded

Outcomes

Outcomes and definitions:

Primary outcome: The primary endpoint is the haemoglobin concentration measured at any time-period during the treatment and follow-up phase

Secondary outcomes: The following endpoints are identified as secondary

For peer review only - http://bmjopen.bmj.com/site/about/guidelines.xhtml

- Anaemia and severe anaemia at baseline, at the time of initial cure assessment and at the time of definitive cure assessment
- White blood cells (WBC) at baseline and at any time during the treatment and follow-up
- Platelets count at baseline and at any time during the treatment and follow-up
- Requirement of blood transfusion during treatment or follow-up (if available)

Anaemia will be defined following the WHO guidelines [10]. The timing of initial cure assessment would typically take place within 28 days of treatment completion but this varies slightly across studies; assessments undertaken between 15 to 60 days will be considered as time of initial cure assessment. Similarly, the timing of definitive cure assessment will vary according to study design and hence assessments made between 5 to 7 months will be considered as 6 months.

Statistical methods

Identification of relevant studies

We searched all the articles indexed in the open-access Infectious Diseases Data Observatory (IDDO) visceral leishmaniasis clinical trials library [11]. The IDDO VL clinical trials library is based on a living systematic review and the database is continually updated every six months in accordance with the Preferred Reporting Items for Systematic-Reviews and Meta-Analyses (PRISMA) guidelines [12]. The trial library indexes publications identified from the following databases: PubMed, Embase, Scopus, Web of Science, Cochrane, clinicaltrials.gov, WHO ICTRP, Global Index Medicus, IMEMR, IMSEAR, and LILACS. For this

For peer review only

For peer review only - http://bmjopen.bmj.com/site/about/guidelines.xhtml

current review, the search includes all clinical trials published between 1st of Jan 1980 and 2nd of May 2021. Details of the search strategy adopted is described elsewhere [12]. Studies indexed in the IDDO VL library will be eligible for inclusion in this review if they meet the inclusion and exclusion criteria outlined above. This review is not limited by language.

Collating IPD

Principal investigators and the corresponding authors of the eligible studies identified from IDDO VL LSR were invited to share IPD. At least two emails will be sent out in case of non-response. Researchers agreeing to the terms and conditions of the submission were invited to upload anonymised IPD to the IDDO repository through a secure web portal [9]. Data in the IDDO VL platform are fully pseudonymised to protect personal information and patient privacy.

Data management

Raw data from individual studies shared with IDDO are currently being standardised using the Clinical Data Interchange Standards Consortium (CDISC) compliant curation standards [13]. Investigators will be further contacted for validation or clarification, if required, and individual study protocols will be requested. On standardisation, the data will be stored in a relational database of several tables containing information on drug regimen, parasitological, clinical, and haematological assessments and therapeutic outcomes.

For peer review only - http://bmjopen.bmj.com/site/about/guidelines.xhtml

Statistical methods for analysis of primary and secondary outcomes

Descriptive summary of the studies included

Summary of included studies will be presented with respect to study location, years of study, study population, duration of follow-up, drug regimen, methodology for diagnosis, supervision of drug administration or treatment adherence (if available), methods and devices used for haematology measurements.

Summary of the participants included

Summary of baseline characteristics of the participants included in the analysis will be presented for each study, by region and overall. The following will be presented: age; weight, parasite grade on enrolment, presence of fever (body temperature > 37.5 degrees Celsius), haemoglobin (or haematocrit), anaemia or severe anaemia as defined using the WHO definitions [10], spleen size, treatment, total mg/kg dose, and supervision of drug administration. The number of available patients will be summarised for all variables, proportions will be used for categorical or binary variables, and mean and standard deviation (or median and interquartile range) will be used for continuous variables.

Analysis of the primary endpoint

Separate linear mixed effect regression models will be undertaken to identify predictors of haemoglobin concentration at baseline, at the time of initial cure assessment and at the time of assessment of definitive cure in a one-stage IPD meta-analysis. The regression models constructed at the time of initial and final cure assessments will adjust for the baseline measurements of haemoglobin as covariates along with the drug regimen.

For peer review only - http://bmjopen.bmj.com/site/about/guidelines.xhtml

If repeated haemoglobin measurements are available for more than 3 time points, the longitudinal haemoglobin profile will be characterised using a linear mixed effects regression model by considering time as a continuous variable. Fractional polynomials will be used to explore any non-linear relationship in the evolution of haemoglobin concentration. If there are few numbers of time-points, then time will be considered as a discrete variable (three time-points: baseline, day 30, and day 180) in the regression model.

The difference between haemoglobin concentration at baseline and at the time of evaluation of definitive cure status (usually at day 180) will be used to quantify the absolute mean disease attributed haematological fall. The difference between haemoglobin concentration at baseline and at the end of the active treatment phase (day 30) will be used to characterise if there is any drug attributed haematological fall.

Further description of candidate predictors and multivariable modelling is described in next.

Candidate predictors and core set

The following variables will be considered for inclusion in the analysis of primary and secondary endpoints.

The following host variables are considered: age, gender, body weight, nutritional status, comorbidity status (such as HIV), and duration of illness prior to study enrolment. Nutritional status in children under 5 years of age will be assessed using standardised age, weight, height and gender specific growth reference standards according to the WHO 2006 recommendations using igrowup Stata package [14] (or equivalent library will in R be used). Anthropometric indicators include weight-for-age (WAZ), height-for-age (HAZ), and weight-

For peer review only - http://bmjopen.bmj.com/site/about/guidelines.xhtml

for-height (WHZ). The nutritional status of a child will be given as a Z-score and classified as stunted, underweight or wasted as defined in the WHO guidelines [14].

The following parasite related baseline factors will be considered: parasite load and information regarding the nature of infection (primary vs. previously treated cases). Any cases described as previously untreated (or "fresh") cases for leishmaniasis will be considered as primary VL.

The following drug related variables will be considered for inclusion in analysis: drug regimen, mg/kg total dose (or target dose), concomitant infections.

The following study or arm level variables will be considered in the analysis of primary and secondary endpoints: geographical region, country, study site, and calendar year of the study conduct.

The following covariates will be examined in the regression model and considered as core predictor set: age, sex, baseline parasite density, HIV co-infection, and geographical region, baseline haematological measurements. These variables along with the drug regimen will form the minimal adjustment set for assessment of other risk factors and will be kept in the regression model regardless of statistical significance [15].

Considerations for multivariable model construction

Multivariable model construction will follow the recommendations of Heinze et al (2017) [16]. Nested models will be compared by assessing the change in log-likelihood estimates and Akaike's information criterion (AIC) will be used for comparing competing non-nested models. The functional form of the continuous variables will be determined using multivariable fractional polynomials [17] or restricted cubic splines [15]. Stability

For peer review only - http://bmjopen.bmj.com/site/about/guidelines.xhtml

investigations will be undertaken to account for uncertainty introduced in multivariable modelling through bootstrap resampling [16].

Handling missing data

To assess the impact of missing data, sensitivity analysis will be performed to see if the main conclusions are affected or not by the exclusion of patients with missing data using multiple imputation [18]. The imputation model will include all the variables in the target analysis and additional auxiliary variables. The target analysis will be carried out in each of the completed (observed plus imputed values) datasets and the estimates will be combined across the imputed datasets using Rubin's combination rules [18].

Sensitivity analyses

Two sensitivity analyses will be carried using resampling techniques to assess model stability. Bootstrap resampling will be used to assess the robustness of the derived estimates and its variance using the recommendations in Heinze et al (2017) [16]. In the second analysis, model will be refitted with one study excluded at a time, and a coefficient of variation around the parameter estimates will be calculated. This would identify any influential studies, that is, studies with unusual results (due to variations in methodology, patient population, and so on) that affect the overall pooled analysis findings.

For peer review only - http://bmjopen.bmj.com/site/about/guidelines.xhtml

Analysis of secondary endpoints

Anaemia and severe anaemia at baseline, at test of initial cure and at end of the study follow-up

A mixed effects logistic regression model will be constructed to identify the predictors associated with anaemia (or severe anaemia) at baseline using a one-stage IPD-MA. Random effects for the study sites will be used to adjust for study-site effect [19]. Potential non-linear relationships between continuous predictors and the outcome will be investigated using multivariable fractional polynomials [20].Similar analysis will be undertaken for anaemia (or severe anaemia) at the time of initial cure, and at the end of the study follow-up. Multivariable model construction will be undertaken as outlined for the primary endpoint.

WBC and Platelets (if data are available)

The distribution of WBC and platelets will be summarised at baseline, at the time of initial cure assessment and at the time of the definitive cure assessment. If data are available, predictors associated with WBC (or platelet counts) will be carried out using a mixed effects linear regression (appropriate transformation will be used if distribution is skewed). Statistical modelling will be undertaken as for the primary endpoint.

Blood transfusion during the study follow-up (if data are available)

Predictors associated with requirement of blood transfusion at any stage during the study period will be identified (if data are available) using a mixed effects logistic regression. Multivariable regression modelling will be undertaken as described for the other endpoints.

For peer review only - http://bmjopen.bmj.com/site/about/guidelines.xhtml

Subgroup analyses

The following two sub-group analyses are planned. Patients living with HIV who are treated for VL typically have worse outcomes and higher mortality risk than those who are not living with HIV [21,22]. A separate sub-group analysis will be carried out among patients with defined VL-HIV coinfections (data permitting). There is a known regional variation in treatment response [23]; a separate analysis could be undertaken for each of the geographical regions.

Risk of bias assessment in included studies

To examine the risk of bias in IPD-MA, the first four domains of the quality In prognosis studies (QUIPS) tool and the first three domains of the prediction model risk of bias assessment tool (PROBAST) will be considered as recommended in Riley et al (2021) [15]. The relevant domains from the QUIPS checklist are study participation, study attrition, prognostic factor measurement, and outcome measurement, and the relevant domains from PROBAST checklist are participant selection, prognostic factors, and outcomes. Two reviewers will independently assess the risk of bias in the studies included in the analysis.

Risk of bias results will be incorporated into analyses by conducting subgroup analyses among studies with overall low risk of bias or by conducting formal interaction analyses with risk of bias responses [24].

Assessment of risk of potential bias in missing studies

Despite best possible efforts, it is anticipated that raw data from all the identified studies will not be available. The characteristics of patient population and study meta-data from the missing studies will be summarised to explore if the missing studies are

For peer review only - http://bmjopen.bmj.com/site/about/guidelines.xhtml

systematically different from the studies that are included in the meta-analysis. A two-stage IPD may be conducted if sufficient details (or any covariate adjustment) are reported in the original studies.

Further development of statistical analysis plan

The main analysis is planned as described above. Modification or additional analyses may be required as the data collection progresses. An updated statistical analysis plan will be available on the IDDO study group website [25].

Software

All the analysis will be carried out using R software or Stata 17 software [26,27]. Use of any other data analysis tools will not change the statistical analysis plan.

Ethics and dissemination

This IPD-MA meets the criteria for waiver of ethical review as defined by the Oxford Tropical Research Ethics Committee (OxTREC) granted to IDDO, as the research consists of secondary analysis of existing anonymised data. Ethical approval was granted to each study included in this pooled analysis by their respective ethics committees. This IPD-MA will address research questions similar to that of included studies. Findings of this IPD-MA will be reported in open-access, peer-reviewed journals following the PRISMA-IPD guidelines [28].

Patient and public involvement

The research questions considered in this IPD-MA is based on a research agenda developed by the global VL research community [29]. The design and development of this IPD-MA were done by the study authors only and no patient was involved at any stage.

For peer review only - http://bmjopen.bmj.com/site/about/guidelines.xhtml

Discussion

Anaemia is the most common haematological manifestation of visceral leishmaniasis (VL). Despite this, the impact of treatment on different haematological parameters remains to be fully understood. The aim of this IPD meta-analysis is to explore the dynamics of different haematological parameters during the treatment and the convalescence phase of the disease. IPD-MA is being used increasingly to explore factors affecting treatment outcomes which otherwise would not be possible through standard aggregate data meta-analysis [30].

This pooled analysis will provide critical information regarding the trajectory of haematological recovery among VL patients. The assessment of the host, parasite and drug determinants that influence the haematological response can provide evidence-based guidance for optimal case management and monitoring drug safety. The IDDO VL LSR, which is a comprehensive library of all published studies since 1980 has been used for identification of the studies eligible for this IPD-MA. A major strength of this study is that data from several studies will be harmonised to a common standard based on an extensive consultation with the VL research community [13], thus allowing us to address some of the methodological sources of heterogeneity. A major challenge is that a substantial proportion of the studies in the IDDO library were conducted prior to the year 2000; the retrieval of data from historical trials is a major challenge.

This IPD-MA will characterise the haematological profile of VL patients at enrolment and at the time-points of initial and definitive cure assessments. Findings of this research will generate important information regarding the evolution of different haematological characteristics.

For peer review only - http://bmjopen.bmj.com/site/about/guidelines.xhtml

Authors' contributions

All authors listed were responsible for the study conceptualisation. AM, PD, PJG, AM, KS wrote the first draft of the manuscript. All authors were involved in reading, revising it critically, editing and approving the final manuscript.

Funding

This work is funded by a Bill & Melinda Gates Foundation grant to the Infectious Diseases Data Observatory, Oxford University, UK (Recipient: Prof. Philippe Guerin; ref: INV-004713).

Conflict of Interest

None

Data Availability

N/A as this is an analysis protocol.

For peer review only

For peer review only - http://bmjopen.bmj.com/site/about/guidelines.xhtml

References

1. Boelaert M, Sundar S. Leishmaniasis. 23rd ed. In: Farrar J, Hotez PJ, Junghanss T, Kang G, Lalloo D, White NJ, editors. Manson's Tropical Diseases. 23rd ed. Elsevier Health Sciences; 2013. pp. 631–651.

2. Goto Y, Cheng J, Omachi S, Morimoto A. Prevalence, severity, and pathogeneses of anemia in visceral leishmaniasis. Parasitol Res. 2017;116: 457–464. doi:10.1007/s00436-016-5313-x

3. Varma N, Naseem S. Hematologic changes in visceral Leishmaniasis/Kala Azar. Indian J Hematol Blood Transfus. 2010;26: 78–82. doi:10.1007/s12288-010-0027-1

4. Kager PA, Rees PH. Haematological investigations in visceral leishmaniasis. Trop Geogr Med. 1986;38: 371–79.

5. Woodruff AW, Topley E, Knight R, Downie CGB. The Anaemia of Kala Azar. Br J Haematol. 1972;22: 319–329. doi:10.1111/j.1365-2141.1972.tb05678.x

6. Thakur CP. Epidemiological, clinical and therapeutic features of Bihar kala-azar (including post kala-azar dermal leishmaniasis). Trans R Soc Trop Med Hyg. 1984;78: 391–398. doi:10.1016/0035-9203(84)90131-7

7. Chulay JD, Bhatt SM, Muigai R, Ho M, Gachihi G, Were JBO, et al. A Comparison of 3 Dosage Regimens of Sodium Stibogluconate in the Treatment of Visceral Leishmaniasis in Kenya. J Infect Dis. 1983;148: 148–155.

8. Shiferaw E, Murad F, Tigabie M, Abebaw M, Alemu T, Abate S, et al. Hematological profiles of visceral leishmaniasis patients before and after treatment of anti-leishmanial drugs at University of Gondar Hospital; Leishmania Research and

For peer review only - http://bmjopen.bmj.com/site/about/guidelines.xhtml

Treatment Center Northwest, Ethiopia. BMC Infect Dis. 2021;21: 1–7. doi:10.1186/s12879-021-06691-7

9. Infectious Diseases Data Observatory. Visceral leishmaniasis Data Inventory. In: iddo.org [Internet]. 2023 [cited 27 Feb 2023]. Available: https://www.iddo.org/document/visceral-leishmaniasis-data-inventory

10. Vitamin and Mineral Nutrition Information System.World Health Organization. Haemoglobin concentrations for the diagnosis of anaemia and assessment of severity. In: Geneva, Switzerland,World Health Organization (WHO/NMH/NHD/MNM/11.1). 2011. doi:2011

11. Infectious Diseases Data Observatory. VL Surveyor. In: www.iddo.org [Internet]. 2020 [cited 17 Sep 2020]. Available: https://www.iddo.org/vlSurveyor/#0

12. Singh-Phulgenda S, Rashan S, Harriss E, Prabin D, Naylor C, Brack M, et al. Infectious Diseases Data Observatory (IDDO) Visceral Leishmaniasis Library of Clinical Therapeutic Studies : A Protocol for a Living Systematic Review of Clinical Studies. In: Research Square PREPRINT (Version 1). 2022. doi:https://doi.org/10.21203/rs.3.rs-1159661/v1 License:

13. Infectious Diseases Data Observatory. New global collaboration developing standards for standardised data collection across the VL research community. 2020 [cited 28 Feb 2023]. Available: https://www.iddo.org/news/new-global-collaboration-developing-standards-standardised-data-collection-across-vl-research

14. WHO Multicentre Growth Reference Study Group. WHO Child Growth Standards: Length/height-for-age, weight-for-age, weight-for-length, weight-for-height and body

For peer review only - http://bmjopen.bmj.com/site/about/guidelines.xhtml

mass index-for-age: Methods and development. . Geneva World Heal Organ. 2006; 312.

15. Riley RD, Moons KGM, Debray TPA. IPD Meta-Analysis for Prognostic Factor Research. In: Richard D. Riley, Jayne F. Tierney LAS, editor. Individual Participant Data Meta-Analysis: A Handbook for Healthcare Research. John Wiley & Sons Ltd; 2021. pp. 421–445. doi:10.1002/9781119333784.ch16

16. Heinze G, Wallisch C, Dunkler D. Variable selection – A review and recommendations for the practicing statistician. Biometrical J. 2018;60: 431–449. doi:10.1002/bimj.201700067

17. Sauerbrei W, Royston P, Binder H. Selection of important variables and determination of functional form for continuous predictors in multivariable model building. Stat Med. 2007;26: 5512–5528. doi:10.1002/sim.3148

18. White IR, Royston P, Wood AM. Multiple imputation using chained equations: Issues and guidance for practice. Stat Med. 2011;30: 377–399. doi:10.1002/sim.4067

19. Glidden D V, Vittinghoff E. Modelling clustered survival data from multicentre clinical trials. Stat Med. 2004;23: 369–88. doi:10.1002/sim.1599

20. Royston P, Sauerbrei W. Multivariable Model-Building: A Pragmatic Approach to Regression Analysis based on Fractional Polynomials for Modelling Continuous Variables. Multivariable Model-Building: A Pragmatic Approach to Regression Analysis based on Fractional Polynomials for Modelling Continuous Variables. John Wiley & Sons, Ltd; 2008. p. pp: 71-98.

21. World Health Organization. WHO guidelines for the treatment of visceral

For peer review only

For peer review only - http://bmjopen.bmj.com/site/about/guidelines.xhtml

leishmaniasis in HIV co-infected patients in East Africa and South-East Asia. World Health OrganizationWorld Health Organization. Geneva, Switzerland; 2022.

22. Singh-Phulgenda S, Rashan S, Harriss E, Dahal P, Naylor C, Brack M, et al. Infectious diseases data observatory (IDDO) visceral leishmaniasis library of clinical therapeutic studies: A protocol for a living systematic review of clinical studies. Wellcome Open Res. 2022;7: 155. doi:10.12688/wellcomeopenres.17739.1

23. Alves F, Bilbe G, Blesson S, Goyal V, Monnerat S, Mowbray C, et al. Recent Development of Visceral Leishmaniasis Treatments: Successes, Pitfalls, and Perspectives. Clin Microbiol Rev. 2018;31: 1–30. doi:10.1128/CMR.00048-18

24. Levis B, Snell K, Takwoingi Y, Collins GS, Moons KG, Reitsma JB, Hooft L, Stewart LA, Tierney JF RR. Evaluating risk of bias and applicability in individual participant data meta-analyses. Advances in Evidence Synthesis: special issue Cochrane Database of Systematic Reviews. 2020. p. (9 Suppl 1). doi:https://doi.org/10.1002/14651858.CD202001

25. Infectious Diseases Data Observatory. Anaemia among patients with visceral leishmaniasis and haematological dynamics post treatment. 2022 [cited 27 Apr 2022]. Available: https://www.iddo.org/anaemia-among-patients-visceral-leishmaniasis-and-haematological-dynamics-post-treatment

26. R Core Team. R: A language and environment for statistical computing. Vienna, Austria; 2021. Available: https://www.r-project.org/.

27. StataCorp. Stata Statistical Software: Release 17. College Station, TX; 2021.

28. Stewart LA, Clarke M, Rovers M, Riley RD, Simmonds M, Stewart G, et al. Preferred

For peer review only

For peer review only - http://bmjopen.bmj.com/site/about/guidelines.xhtml

reporting items for a systematic review and meta-analysis of individual participant data: The PRISMA-IPD statement. JAMA - J Am Med Assoc. 2015;313: 1657–1665. doi:10.1001/jama.2015.3656

29. Infectious Diseases Data Observatory. Visceral leishmaniasis research agenda. In: 2019 [Internet]. [cited 28 Feb 2023]. Available: https://www.iddo.org/vl/research/research-agenda

30. Riley RD, Lambert PC, Abo-Zaid G. Meta-analysis of individual participant data: Rationale, conduct, and reporting. BMJ. 2010;340: 521–525. doi:10.1136/bmj.c221

For peer review only

For peer review only - http://bmjopen.bmj.com/site/about/guidelines.xhtml

BMJ Open

Haematological dynamics following treatment of visceral leishmaniasis: a protocol for systematic review and individual participant data (IPD) meta-analysis

Journal:	*BMJ Open*
Manuscript ID	bmjopen-2023-074841.R1
Article Type:	Protocol
Date Submitted by the Author:	11-Oct-2023

Complete List of Authors:	Munir, Abdalla; Infectious Diseases Data Observatory (IDDO); University of Oxford, Centre for Tropical Medicine and Global Health, Nuffield Department of Medicine<br>Dahal, Prabin; University of Oxford, Centre for Tropical Medicine and Global Health, Nuffield Department of Medicine<br>Kumar, Rishikesh; Rajendra Memorial Research Institute of Medical Sciences (RMRIMS)<br>Singh-Phulgenda, Sauman; Infectious Diseases Data Observatory (IDDO); University of Oxford, Centre for Tropical Medicine and Global Health, Nuffield Department of Medicine<br>Siddiqui, Niyamat; Rajendra Memorial Research Institute of Medical Sciences (RMRIMS)<br>Naylor, Caitlin; Infectious Diseases Data Observatory (IDDO); University of Oxford, Centre for Tropical Medicine and Global Health, Nuffield Department of Medicine<br>Wilson, James; Infectious Diseases Data Observatory (IDDO); University of Oxford, Centre for Tropical Medicine and Global Health, Nuffield Department of Medicine<br>Buck, Gemma; Infectious Diseases Data Observatory (IDDO); University of Oxford, Centre for Tropical Medicine and Global Health, Nuffield Department of Medicine<br>Rahi, Manju; Indian Council of Medical Research (ICMR)<br>Alves, Fabiana; Drugs for Neglected Disease Initiative<br>Malaviya, Paritosh; Banaras Hindu University, Infectious Disease Research Laboratory, Department of Medicine, Institute of Medical Sciences<br>Sundar, Shyam; Banaras Hindu University, Infectious Disease Research Laboratory, Department of Medicine, Institute of Medical Sciences<br>Ritmeijer, Koert; Médecins Sans Frontières<br>Stepniewska, Kasia; Infectious Diseases Data Observatory (IDDO); University of Oxford, Centre for Tropical Medicine and Global Health, Nuffield Department of Medicine<br>Pandey, Krishna; Rajendra Memorial Research Institute of Medical Sciences (RMRIMS)<br>Guérin, Philippe J.; Infectious Diseases Data Observatory (IDDO); University of Oxford, Centre for Tropical Medicine and Global Health, Nuffield Department of Medicine<br>Musa, Ahmed; University of Khartoum, Institute of Endemic Diseases

**Primary Subject Heading**:	Infectious diseases
Secondary Subject Heading:	Public health
Keywords:	Anaemia < HAEMATOLOGY, EPIDEMIOLOGY, INFECTIOUS DISEASES, Tropical medicine < INFECTIOUS DISEASES

SCHOLARONE™ Manuscripts

Haematological dynamics following treatment of visceral leishmaniasis: a protocol for systematic review and individual participant data (IPD) meta-analysis

Abdalla Munir[1,2,3], Prabin Dahal[1,2§], Rishikesh Kumar[4], Sauman Singh-Phulgenda[1,2], Niyamat Ali Siddiqui[4], Caitlin Naylor[1,2], James Wilson[1,2], Gemma Buck[1,2], Manju Rahi[5], Fabiana Alves[6], Paritosh Malaviya[7], Shyam Sundar[7], Koert Ritmeijer[8], Kasia Stepniewska[1,2], Krishna Pandey[4], Phillipe J Guerin[1,2§], Ahmed Musa[3§]

[1]Infectious Diseases Data Observatory (IDDO), Oxford, UK

[2]Centre for Tropical Medicine and Global Health, Nuffield Department of Clinical Medicine, University of Oxford, Oxford, UK

[3] Institute of Endemic Diseases, University of Khartoum, Khartoum, Sudan

[4]Rajendra Memorial Research Institute of Medical Sciences (RMRIMS), Patna, India

[5]Indian Council of Medical Research (ICMR), New Delhi, India

[6]Drugs for Neglected Diseases initiative, Geneva, Switzerland

[7]Infectious Disease Research Laboratory, Department of Medicine, Institute of Medical Sciences, Banaras Hindu University, Varanasi, India

[8]Médecins Sans Frontières, Amsterdam, The Netherlands

[§]Correspondence: prabin.dahal@ndm.ox.ac.uk & musaam2003@yahoo.co.uk

For peer review only - http://bmjopen.bmj.com/site/about/guidelines.xhtml

Authors' email:

AbM abdalla.munir@iddo.org

PD prabin.dahal@iddo.org

RK rishikeshkumar@live.in

SSP sauman.singh@iddo.org

NAS niyamatalisiddiqui@yahoo.com

CN caitlin.naylor@iddo.org

JW james.wilson@iddo.org

GB gemma.buck@iddo.org

MR drmanjurahi@gmail.com

FA falves@dndi.org

PM paritosh_malaviya@yahoo.com

SS drshyamsundar@hotmail.com

KR koert.ritmeijer@amsterdam.msf.org

KS kasia.stepniewska@iddo.org

KP drkrishnapandey@yahoo.com

PJG philippe.guerin@iddo.org

AhM musaam2003@yahoo.co.uk

For peer review only

Abstract

Introduction: Visceral leishmaniasis (VL) is a parasitic disease with an estimated 30,000 new cases occurring annually. Despite anaemia being a common haematological manifestation of VL, the evolution of different haematological characteristics following treatment remains poorly understood. An individual participant data meta-analysis (IPD-MA) is planned to characterise the haematological dynamics in VL patients.

Methods and analysis: The Infectious Diseases Data Observatory (IDDO) VL data platform is a global repository of individual participant data from therapeutic studies identified through a systematic search of published literature (PROSPERO registration: CRD42021284622). The platform currently holds datasets from clinical trials standardised to a common data format. Corresponding authors and principal investigators of the studies indexed in the IDDO VL data platform meeting the eligibility criteria for inclusion in this IPD-MA were invited to be part of the collaborative IPD-MA. Mixed effects multivariable regression models will be constructed to identify determinants of haematological parameters by taking clustering within study-sites into account.

Ethics and dissemination: This IPD-MA meets the criteria for waiver of ethical review as defined by the Oxford Tropical Research Ethics Committee (OxTREC) granted to IDDO, as the research consists of secondary analysis of existing anonymised data (exempt granted on 29th March 2023, OxTREC REF: Infectious Diseases Data Observatory (IDDO)). Ethics approval was granted by the ICMR-Rajendra Memorial Research Institute of Medical Sciences ethics committee (Letter no: RMRI/EC/30/2022) on 04-07-2022. The results of this

For peer review only

For peer review only - http://bmjopen.bmj.com/site/about/guidelines.xhtml

analysis will be disseminated at conferences, IDDO website and peer-reviewed publication in open-access journals. Findings of this research will be critically important for the control programmes at regional/global levels, policy makers and groups developing new VL treatment.

PROSPERO registration: The systematic review component of the IPD-MA has the following PROSPERO registration: CRD42021284622.

Keywords

Visceral Leishmaniasis; Kala-azar; Statistical Analysis Plan, meta-analysis, individual participant data; IPD-MA; anaemia, haemoglobin; platelets, white blood cells, red blood cells

Word count: 2841 (main text); abstract (265)

For peer review only

For peer review only - http://bmjopen.bmj.com/site/about/guidelines.xhtml

Strengths and limitations of this study

- An individual participant data meta-analysis (IPD-MA) is proposed to characterise the evolution of haemoglobin and other haematological parameters during the study period; this will overcome the limitations due to sample size issues.

- The identification of studies has been made through a comprehensive literature search of all published studies since 1980 with a pre-defined inclusion-exclusion criterion.

- A major strength of this IPD-MA is that it uses individual participant data hosted at Infectious Diseases Data Observatory which has harmonised raw data to a common standard based on an extensive consultation with the VL research community.

- Retrieval of data from trials published prior to 2000 can be a major challenge.

For peer review only

For peer review only - http://bmjopen.bmj.com/site/about/guidelines.xhtml

Introduction

Anaemia is a common haematological manifestation of Visceral Leishmaniasis (VL).[1] In patients with VL, anaemia can arise due to one or more associated factors: sequestration and haemolysis of red blood cells in the spleen associated with hypersplenism, bone marrow suppression caused by nutritional deficiencies such as iron, vitamin B12 and folate deficiencies, or due to clotting dysfunction leading to blood loss [1–5]. At the time of clinical diagnosis, haemoglobin levels are often around 7–10 g/dL but can be as low as 4 g/dL.[1] The severity of anaemia depends on the duration of clinical illness and can be exacerbated by comorbidities and iron deficiency [6, 7].

After treatment with an antileishmanial drug, haematological improvement generally occurs within a few weeks with significant recovery expected within 4-6 weeks [3]. The trajectory of haematological recovery may be affected by patients' age or initial parasite load [8], but the influence of drug regimen and other patient characteristics remains poorly understood. Haematological safety of the VL treatment remains an important concern [9]. Therefore, characterisation of haematological profile and identification of drivers associated with haematological recovery can help in optimal case management. The present individual participant data meta-analysis (IPD-MA) aimed to characterise the haematological changes in VL patients after treatment, and understand the role of host, parasite and drug related characteristics.

Objectives

The objectives of this IPD-MA are:

1. To identify the determinants of haemoglobin concentration at enrolment, at initial cure assessment and at definitive cure assessment

For peer review only

For peer review only - http://bmjopen.bmj.com/site/about/guidelines.xhtml

2. To characterise the haemoglobin dynamics following treatment
3. To identify the determinants of anaemia or severe anaemia at enrolment, at initial cure assessment and at definitive cure assessment
4. To describe white blood cells and platelets dynamics following treatment (data permitting)
5. To identify predictors of blood transfusion during treatment or follow-up (if available)

Methods and analysis: Patients, interventions and outcomes

Elements of the research aim (PICOT)

Population: Any patient enrolled in a prospective efficacy studies with a confirmed or suspected diagnosis of VL defined by serological and/or parasitological testing.

Interventions: Any antileishmanial therapy.

Comparator: Not restricted by the use of a comparator drug.

Outcome: At least one of the following outcomes reported: anaemia (or haemoglobin measurements) at enrolment, anaemia and other haematological measurements at any post-baseline time-points.

Time: Studies published on or after 1980.

Criteria for study eligibility

Studies in the IDDO VL data platform [10] will be deemed eligible for the purpose of this IPD-MA if they meet the following criteria:

For peer review only - http://bmjopen.bmj.com/site/about/guidelines.xhtml

- Prospective efficacy studies on patients with confirmed or suspected VL either using microscopy/serology/molecular method (i.e. clinical diagnosis followed by a confirmatory method)
- Information is available on treatment regimen including drug, dose, and duration of regimen
- Data on anaemia (or haemoglobin/haematocrit concentration) measured at enrolment

Desirable criteria

- Methodology used for haematological quantification, e.g. name of device used
- Information regarding if blood transfusion were given before or after treatment initiation
- Information on co-administration of iron supplement

Criteria for participant eligibility

The minimum information required for participants from each of the identified studies for inclusion in the IPD-MA analysis are listed below:

- Details of antileishmanial treatment(s) administered
- Baseline information on age and gender
- Outcome is recorded

Outcomes

Outcomes and definitions:

For peer review only

For peer review only - http://bmjopen.bmj.com/site/about/guidelines.xhtml

Primary outcome: The primary outcome is the haemoglobin concentration measured at any time-period during the treatment and follow-up phase.

Secondary outcomes: The following endpoints are identified as secondary:

- Anaemia and severe anaemia at baseline, at the time of initial cure assessment and at the time of definitive cure assessment
- White blood cells (WBC) at baseline and at any time during the treatment and follow-up
- Platelets count at baseline and at any time during the treatment and follow-up
- Requirement of blood transfusion during treatment or follow-up (if available)

Anaemia will be defined following the WHO guidelines [11]. The timing of initial cure assessment would typically take place within 28 days of treatment completion but this varies slightly across studies; assessments undertaken between 15 to 60 days will be considered as time of initial cure assessment. Similarly, the timing of definitive cure assessment will vary according to study design and hence assessments made between 5 to 7 months will be considered as 6 months.

Statistical methods

Identification of relevant studies using IDDO VL library

We searched all the articles indexed in the open-access Infectious Diseases Data Observatory visceral leishmaniasis clinical trials library (IDDO VL library).[12] The IDDO VL library is continually updated and follows the Preferred Reporting Items for Systematic-Reviews and Meta-Analyses (PRISMA) guidelines [13]. The IDDO VL library indexes

For peer review only - http://bmjopen.bmj.com/site/about/guidelines.xhtml

publications identified from the following databases: PubMed, Embase, Scopus, Web of Science, Cochrane, clinicaltrials.gov, WHO ICTRP, Global Index Medicus, IMEMR, IMSEAR, and LILACS. For this current review, the search includes all clinical trials published between 1st of Jan 1980 and 2nd of May 2021. Details of the search strategy adopted is described elsewhere [13]. The search details are presented in online supplemental file 1. Studies indexed in the IDDO VL library will be eligible for inclusion in this review if they meet the inclusion and exclusion criteria outlined above. This review is not limited by language.

Collating IPD: IDDO VL data platform

Principal investigators and the corresponding authors of the eligible studies identified from IDDO VL library were invited to share IPD. At least two emails will be sent out in case of non-response. Researchers agreeing to the terms and conditions of the submission were invited to upload anonymised IPD to the IDDO repository through a secure web portal [10]. Data in the IDDO VL platform are fully pseudonymised to protect personal information and patient privacy.

Data management

Raw data from individual studies shared with IDDO are currently being standardised using the Clinical Data Interchange Standards Consortium (CDISC) compliant curation standards [14]. Investigators will be further contacted for validation or clarification, if required, and individual study protocols will be requested. On standardisation, the data will be stored in a relational database of several tables containing information on drug regimen, parasitological, clinical, and haematological assessments and therapeutic outcomes.

For peer review only - http://bmjopen.bmj.com/site/about/guidelines.xhtml

Statistical methods for analysis of primary and secondary outcomes

Descriptive summary of the studies included

Summary of included studies will be presented with respect to study location, years of study, study population, duration of follow-up, drug regimen, methodology for diagnosis, supervision of drug administration or treatment adherence (if available), methods and devices used for haematology measurements.

Summary of the participants included

Summary of baseline characteristics of the participants included in the analysis will be presented for each study, by region and overall. The following will be presented: age; weight, parasite grade on enrolment, presence of fever (body temperature > 37.5 degrees Celsius), haemoglobin (or haematocrit), anaemia or severe anaemia as defined using the WHO definitions [11], spleen size, treatment, total mg/kg dose, and supervision of drug administration. The number of available patients will be summarised for all variables, proportions will be used for categorical or binary variables, and mean and standard deviation (or median and interquartile range) will be used for continuous variables.

Analysis of the primary endpoint

Separate linear mixed effect regression models will be undertaken to identify predictors of haemoglobin concentration at baseline, at the time of initial cure assessment and at the time of assessment of definitive cure in a one-stage IPD meta-analysis. The regression models constructed at the time of initial and final cure assessments will adjust for the baseline measurements of haemoglobin as covariates along with the drug regimen.

For peer review only - http://bmjopen.bmj.com/site/about/guidelines.xhtml

If repeated haemoglobin measurements are available for more than 3 time-points, the longitudinal haemoglobin profile will be characterised using a linear mixed effects regression model by considering time as a continuous variable. Fractional polynomials will be used to explore any non-linear relationship in the evolution of haemoglobin concentration. If there are few numbers of time-points, then time will be considered as a discrete variable (three time-points: baseline, day 30, and day 180) in the regression model. If frequent data is available, then the haemoglobin concentration will be summarised at weekly time-points during the follow-up period.

The difference between haemoglobin concentration at baseline and at the time of evaluation of definitive cure status (usually at day 180) will be used to quantify the absolute mean disease attributed haematological fall. The difference between haemoglobin concentration at baseline and at the end of the active treatment phase (day 30) will be used to gauge if there is any drug attributed haematological fall.

Further description of candidate predictors and multivariable modelling is described next.

Candidate predictors and core set

The following variables will be considered for inclusion in the analysis of primary and secondary endpoints.

The following host variables are considered: age, gender, body weight, nutritional status, comorbidity status (such as HIV), and duration of illness prior to study enrolment. Nutritional status in children under 5 years of age will be assessed using standardised age, weight, height and gender specific growth reference standards according to the WHO 2006

For peer review only

For peer review only - http://bmjopen.bmj.com/site/about/guidelines.xhtml

recommendations using igrowup Stata package (or equivalent library will in R be used) [15]. Anthropometric indicators include weight-for-age (WAZ), height-for-age (HAZ), and weight-for-height (WHZ). The nutritional status of a child will be given as a Z-score and classified as stunted, underweight or wasted as defined in the WHO guidelines [15].

The following parasite related baseline factors will be considered: parasite load and information regarding the nature of infection (primary vs. previously treated cases). Any cases described as previously untreated (or “fresh”) cases for leishmaniasis will be considered as primary VL.

The following drug related variables will be considered for inclusion in analysis: drug regimen, mg/kg total dose (or target dose), concomitant infections.

The following study or arm level variables will be considered in the analysis of primary and secondary endpoints: geographical region, country, study site, and calendar year of the study conduct.

The following covariates will be examined in the regression model and considered as core predictor set: age, sex, baseline parasite density, HIV co-infection, geographical region, baseline haematological measurements. These variables along with the drug regimen will form the minimal adjustment set for assessment of other risk factors and will be kept in the regression model regardless of statistical significance[16].

Considerations for multivariable model construction

Multivariable model construction will follow the recommendations of Heinze et al (2017) [17]. Nested models will be compared by assessing the change in log-likelihood estimates and Akaike’s information criterion (AIC) will be used for comparing competing

For peer review only - http://bmjopen.bmj.com/site/about/guidelines.xhtml

non-nested models. The functional form of the continuous variables will be determined using multivariable fractional polynomials [18] or restricted cubic splines [16]. Stability investigations will be undertaken to account for uncertainty introduced in multivariable modelling through bootstrap resampling [17].

Handling missing data

To assess the impact of missing data, sensitivity analysis will be performed to see if the main conclusions are affected by the exclusion of patients with missing data using multiple imputation [19]. The imputation model will include all the variables in the target analysis and additional auxiliary variables. The target analysis will be carried out in each of the completed (observed plus imputed) datasets and the estimates will be combined across the imputed datasets using Rubin's combination rules [19].

Sensitivity analyses

Two sensitivity analyses will be carried out using resampling techniques to assess model stability. Bootstrap resampling will be used to assess the robustness of the derived estimates and its variance using the recommendations in Heinze et al (2017) [17]. In the second analysis, model will be refitted with one study excluded at a time, and a coefficient of variation around the parameter estimates will be calculated. This would identify any influential studies, that is, studies with unusual results (due to variations in underlying methodology to measure outcomes, patient population, and so on) that affect the overall pooled analysis findings.

For peer review only

Analysis of secondary endpoints

Anaemia and severe anaemia at baseline, at test of initial cure and at end of the study follow-up

A mixed effects logistic regression model will be constructed to identify the predictors associated with anaemia (or severe anaemia) at baseline using a one-stage IPD-MA. Random effects for the study sites will be used to adjust for study-site effect [20]. Potential non-linear relationships between continuous predictors and the outcome will be investigated using multivariable fractional polynomials [21]. Similar analysis will be undertaken for anaemia (or severe anaemia) at the time of initial cure, and at the end of the study follow-up. Multivariable model construction will be undertaken as outlined for the primary endpoint.

WBC and Platelets (if data are available)

The distribution of WBC and platelets will be summarised at baseline, at the time of initial cure assessment and at the time of the definitive cure assessment. If data are available, predictors associated with WBC (or platelet counts) will be carried out using a mixed effects linear regression (appropriate transformation will be used if distribution is skewed). Statistical modelling will be undertaken as for the primary endpoint.

Blood transfusion during the study follow-up (if data are available)

Predictors associated with requirement of blood transfusion at any stage during the study period will be identified (if data are available) using a mixed effects logistic regression. Multivariable regression modelling will be undertaken as described for the other endpoints.

For peer review only

For peer review only - http://bmjopen.bmj.com/site/about/guidelines.xhtml

Subgroup analyses

Patients living with HIV who are treated for VL typically have worse outcomes and higher mortality risk than those who are not living with HIV [22]. A separate sub-group analysis will be carried out among patients with defined VL-HIV coinfections (data permitting). There is a known regional variation in treatment response in VL along with differences in patient characteristics and treatment guidelines [23]. Therefore, a separate analysis will be undertaken within each geographical region to construct the univariable and multivariable regression models for the primary and secondary outcomes.

Longitudinal haemoglobin profile will be stratified by the transfusion status at any stage of the study.

Risk of bias assessment in included studies

To examine the risk of bias in IPD-MA, the first four domains of the quality In prognosis studies (QUIPS) tool and the first three domains of the prediction model risk of bias assessment tool (PROBAST) will be considered as recommended in Riley et al (2021)[16]. The relevant domains from the QUIPS checklist are study participation, study attrition, prognostic factor measurement, and outcome measurement, and the relevant domains from PROBAST checklist are participant selection, prognostic factors, and outcomes. Two reviewers will independently assess the risk of bias in the studies included in the analysis.

Risk of bias results will be incorporated into analyses by conducting subgroup analyses among studies with overall low risk of bias or by conducting formal interaction analyses with risk of bias responses [24].

For peer review only - http://bmjopen.bmj.com/site/about/guidelines.xhtml

For peer review only

Assessment of risk of potential bias in missing studies

Despite best possible efforts, it is anticipated that raw data from all the identified studies will not be available. The characteristics of patient population and study meta-data from the missing studies will be summarised to explore if the missing studies are systematically different from the studies that are included in the IPD-MA. A two-stage IPD-MA will be considered if sufficient details (or any covariate adjustment) are reported in the original studies.

Software

All the analysis will be carried out using R software or Stata 17 software [25, 26]. Use of any other data analysis tools will not change the statistical analysis plan.

Dissemination plans

Ethics and dissemination

This IPD-MA meets the criteria for waiver of ethical review as defined by the Oxford Tropical Research Ethics Committee (OxTREC) granted to IDDO, as the research consists of secondary analysis of existing anonymised data. Ethics approval was granted by the ICMR-Rajendra Memorial Research Institute of Medical Sciences ethics committee (Letter no: RMRI/EC/30/2022) on 04-07-2022. Ethical approval was granted to each study included in this pooled analysis by their respective ethics committees. This IPD-MA will address research questions similar to that of included studies. Findings of this IPD-MA will be reported in open-access, peer-reviewed journals following the PRISMA-IPD guidelines [27].

For peer review only

For peer review only - http://bmjopen.bmj.com/site/about/guidelines.xhtml

352 **Patient and public involvement**

353 The design and development of this IPD-MA were done by the study authors only and no
354 patient was involved at any stage. Patients and/or the public were not involved in the
355 design, or conduct, or reporting or dissemination plans of this research. The research
356 questions considered in this IPD-MA is based on a research agenda developed by the global
357 VL research community [28].

358 **Further development of statistical analysis plan**

359 The main analysis is planned as described above. Modification or additional analyses
360 may be required as the data collection progresses. Any modifications of the analysis will be
361 documented and made publicly available on the IDDO study group website [29].

For peer review only

For peer review only - http://bmjopen.bmj.com/site/about/guidelines.xhtml

Discussion

Anaemia is a common haematological manifestation of visceral leishmaniasis. Despite this, the impact of treatment on different haematological parameters remains to be fully understood. The aim of this IPD meta-analysis is to explore the dynamics of different haematological parameters during the treatment and the convalescence phase of the disease. IPD-MA is being used increasingly to explore factors affecting treatment outcomes which otherwise would not be possible through standard aggregate data meta-analysis [30].

This IPD-MA will provide critical information regarding the trajectory of haematological recovery among VL patients. The assessment of the host, parasite and drug determinants that influence the haematological response can provide evidence-based guidance for optimal case management and monitoring drug safety. The IDDO VL library, which is a comprehensive library of all published studies since 1980 has been used for identification of the studies eligible for this IPD-MA which has led to the construction of the IDDO VL data platform. A major strength of this study is that data from several studies will be harmonised to a common standard (in the IDDO VL data platform) based on an extensive consultation with the VL research community [14], thus allowing us to address some of the methodological sources of heterogeneity.

A major challenge is that a substantial proportion of the studies in the IDDO library were conducted prior to the year 2000; the retrieval of data from historical trials is a major challenge [31, 32]. Another limitation is that the exploration of the predictors of anaemia and severe anaemia at baseline will be influenced by the underlying eligibility criteria adopted in clinical trials as clinical trials frequently exclude patients with very low haemoglobin at presentation [33]. Compared to non-pregnant patients, pregnant women

For peer review only - http://bmjopen.bmj.com/site/about/guidelines.xhtml

are more at an increased risk of requiring blood transfusion [34]. However, pregnancy is often adopted as exclusion criteria in VL trials [33] and the haematological consequences in this important patient group couldn't be explore despite the clear recognition of anaemia and transfusion needs.

This IPD-MA will characterise the haematological profile of VL patients at enrolment and at the time-points of initial and definitive cure assessments. Findings of this research will generate important information regarding the evolution of different haematological characteristics.

For peer review only

For peer review only - http://bmjopen.bmj.com/site/about/guidelines.xhtml

Authors' contributions

Study conception: AbM, PD, RK, SS-P, NAS, CN, JW, GB, MR, FA, PM, SS, KR, KS, KP, PJG and AhM. Project supervision: PD, AbM, KS, and PJG. Methodology: PD, NAS, SS-P, JW, FA, PJG and KS. Data curation: AbM, SS-P, PM, JW, PD and GB. Project administration: SS-P and CN. Funding acquisition: PJG. Resources: SS-P, FA and PJG. Writing-original draft: AbM, RK, SSP, KS, PJG, AhM, and PD. Writing- review and editing: All authors were involved in reading and critical revision of the initial draft and approved the final manuscript.

Funding

This work is funded by a Bill & Melinda Gates Foundation grant to the Infectious Diseases Data Observatory, Oxford University, UK (Recipient: Prof. Philippe Guerin; ref: INV-004713). Funding agency had no role in developing the protocol.

Conflict of Interest

None

Data Availability

N/A as this is an analysis protocol.

Patient consent for publication

Not required.

Supplemental file

Supplemental file 1: Search Details

For peer review only

For peer review only - http://bmjopen.bmj.com/site/about/guidelines.xhtml

References

1. Boelaert M, Sundar S. Leishmaniasis. In: Farrar J, Hotez PJ, Junghanss T, Kang G, Lalloo D, White NJ, editors. Manson's Tropical Diseases. 23rd edition. Elsevier Health Sciences; 2013. p. 631–51.

2. Goto Y, Cheng J, Omachi S, Morimoto A. Prevalence, severity, and pathogeneses of anemia in visceral leishmaniasis. Parasitol Res. 2017;116:457–64.

3. Varma N, Naseem S. Hematologic changes in visceral Leishmaniasis/Kala Azar. Indian J Hematol Blood Transfus. 2010;26:78–82.

4. Kager PA, Rees PH. Haematological investigations in visceral leishmaniasis. Trop Geogr Med. 1986;38:371–9.

5. Woodruff AW, Topley E, Knight R, Downie CGB. The Anaemia of Kala Azar. Br J Haematol. 1972;22:319–29.

6. Thakur CP. Epidemiological, clinical and therapeutic features of Bihar kala-azar (including post kala-azar dermal leishmaniasis). Trans R Soc Trop Med Hyg. 1984;78:391–8.

7. Chulay JD, Bhatt SM, Muigai R, Ho M, Gachihi G, Were JBO, et al. A Comparison of 3 Dosage Regimens of Sodium Stibogluconate in the Treatment of Visceral Leishmaniasis in Kenya. J Infect Dis. 1983;148:148–55.

8. Shiferaw E, Murad F, Tigabie M, Abebaw M, Alemu T, Abate S, et al. Hematological profiles of visceral leishmaniasis patients before and after treatment of anti-leishmanial drugs at University of Gondar Hospital; Leishmania Research and Treatment Center Northwest, Ethiopia. BMC Infect Dis. 2021;21:1–7.

For peer review only

For peer review only - http://bmjopen.bmj.com/site/about/guidelines.xhtml

9. Singh-Phulgenda S, Dahal P, Ngu R, Maguire BJ, Hawryszkiewycz A, Rashan S, et al. Serious adverse events following treatment of visceral leishmaniasis: A systematic review and meta-analysis. PLoS Negl Trop Dis. 2021;15:e0009302.

10. Infectious Diseases Data Observatory. Visceral leishmaniasis Data Inventory. iddo.org. 2023. https://www.iddo.org/document/visceral-leishmaniasis-data-inventory. Accessed 27 Feb 2023.

11. Vitamin and Mineral Nutrition Information System.World Health Organization. Haemoglobin concentrations for the diagnosis of anaemia and assessment of severity. Geneva, Switzerland,World Health Organization (WHO/NMH/NHD/MNM/11.1). 2011.

12. Infectious Diseases Data Observatory. VL Surveyor. www.iddo.org. 2020. https://www.iddo.org/vlSurveyor/#0. Accessed 17 Sep 2020.

13. Singh-Phulgenda S, Rashan S, Harriss E, Dahal P, Naylor C, Brack M, et al. Infectious diseases data observatory (IDDO) visceral leishmaniasis library of clinical therapeutic studies: A protocol for a living systematic review of clinical studies. Wellcome Open Res. 2022;7 May:155.

14. Infectious Diseases Data Observatory. New global collaboration developing standards for standardised data collection across the VL research community. 2020. https://www.iddo.org/news/new-global-collaboration-developing-standards-standardised-data-collection-across-vl-research. Accessed 28 Feb 2023.

15. WHO Multicentre Growth Reference Study Group. WHO Child Growth Standards: Length/height-for-age, weight-for-age, weight-for-length, weight-for-height and body mass index-for-age: Methods and development. . Geneva World Heal Organ. 2006;:312.

For peer review only

For peer review only - http://bmjopen.bmj.com/site/about/guidelines.xhtml

16. Riley RD, Moons KGM, Debray TPA. IPD Meta-Analysis for Prognostic Factor Research. In: Richard D. Riley, Jayne F. Tierney LAS, editor. Individual Participant Data Meta-Analysis: A Handbook for Healthcare Research. John Wiley & Sons Ltd; 2021. p. 421–45.

17. Heinze G, Wallisch C, Dunkler D. Variable selection – A review and recommendations for the practicing statistician. Biometrical J. 2018;60:431–49.

18. Sauerbrei W, Royston P, Binder H. Selection of important variables and determination of functional form for continuous predictors in multivariable model building. Stat Med. 2007;26:5512–28.

19. White IR, Royston P, Wood AM. Multiple imputation using chained equations: Issues and guidance for practice. Stat Med. 2011;30:377–99.

20. Glidden D V, Vittinghoff E. Modelling clustered survival data from multicentre clinical trials. Stat Med. 2004;23:369–88.

21. Royston P, Sauerbrei W. Multivariable Model-Building: A Pragmatic Approach to Regression Analysis based on Fractional Polynomials for Modelling Continuous Variables. In: Multivariable Model-Building: A Pragmatic Approach to Regression Analysis based on Fractional Polynomials for Modelling Continuous Variables. John Wiley & Sons, Ltd; 2008. p. pp: 71-98.

22. World Health Organization. WHO guidelines for the treatment of visceral leishmaniasis in HIV co-infected patients in East Africa and South-East Asia. Geneva, Switzerland; 2022.

23. Alves F, Bilbe G, Blesson S, Goyal V, Monnerat S, Mowbray C, et al. Recent Development of Visceral Leishmaniasis Treatments: Successes, Pitfalls, and Perspectives. Clin Microbiol Rev. 2018;31:1–30.

For peer review only - http://bmjopen.bmj.com/site/about/guidelines.xhtml

24. Levis B, Snell K, Takwoingi Y, Collins GS, Moons KG, Reitsma JB, Hooft L, Stewart LA, Tierney JF RR. Evaluating risk of bias and applicability in individual participant data meta-analyses. In: Advances in Evidence Synthesis: special issue Cochrane Database of Systematic Reviews. 2020. p. (9 Suppl 1).

25. R Core Team. R: A language and environment for statistical computing. 2021.

26. StataCorp. Stata Statistical Software: Release 17. 2021.

27. Stewart LA, Clarke M, Rovers M, Riley RD, Simmonds M, Stewart G, et al. Preferred reporting items for a systematic review and meta-analysis of individual participant data: The PRISMA-IPD statement. JAMA - J Am Med Assoc. 2015;313:1657–65.

28. Infectious Diseases Data Observatory. Visceral leishmaniasis research agenda. 2019. https://www.iddo.org/vl/research/research-agenda. Accessed 28 Feb 2023.

29. Infectious Diseases Data Observatory. Anaemia among patients with visceral leishmaniasis and haematological dynamics post treatment. 2022. https://www.iddo.org/anaemia-among-patients-visceral-leishmaniasis-and-haematological-dynamics-post-treatment. Accessed 27 Apr 2022.

30. Riley RD, Lambert PC, Abo-Zaid G. Meta-analysis of individual participant data: Rationale, conduct, and reporting. BMJ. 2010;340:521–5.

31. Vines TH, Albert AYK, Andrew RL, Débarre F, Bock DG, Franklin MT, et al. The availability of research data declines rapidly with article age. Curr Biol. 2014;24:94–7.

32. Nevitt SJ, Marson AG, Davie B, Reynolds S, Williams L, Smith CT. Exploring changes over time and characteristics associated with data retrieval across individual participant data meta-analyses: Systematic review. BMJ. 2017;357.

For peer review only

For peer review only - http://bmjopen.bmj.com/site/about/guidelines.xhtml

33. Dahal P, Singh-Phulgenda S, Naylor C, Brack M, Chatterjee M, Alves F, et al. Design, conduct, analysis, and reporting of therapeutic efficacy studies in Visceral Leishmaniasis: A systematic review of published reports, 2000-2021 Journal: Under Rev. 2023. https://doi.org/https://doi.org/10.1101/2023.09.06.23295148.

34. Pekelharing JE, Gatluak F, Harrison T, Maldonado F, Siddiqui R, Ritmeijer K. Outcomes of visceral leishmaniasis in pregnancy : A retrospective cohort study from South Sudan. PLoS Negl Trop Dis. 2020;14:e0007992.

For peer review only

For peer review only - http://bmjopen.bmj.com/site/about/guidelines.xhtml

Example of Search Strategies: The dates will be different for future searches

Pubmed

((((((((randomized controlled trial[pt] OR controlled clinical trial[pt] OR randomized[tiab] OR placebo[tiab] OR clinical trials as topic[mesh:noexp] OR randomly[tiab] OR trial[ti] NOT (animals[mh] NOT humans [mh]))) AND Clinical Trial[ptyp])) AND (((("Leishmaniasis, Visceral"[Mesh]) OR visceral leishmaniasis[Title/Abstract]) OR kala azar[Title/Abstract]) OR black fever[Title/Abstract]))) OR (((((((((((((("Pentamidine"[Mesh]) OR "liposomal amphotericin B" [Supplementary Concept]) OR "Amphotericin B"[Mesh]) OR "Paromomycin"[Mesh]) OR "miltefosine" [Supplementary Concept]) OR "Sodium"[Mesh]) OR "8-aminoquinoline" [Supplementary Concept]) OR "Ketoconazole"[Mesh]) OR "Azoles"[Mesh]) OR "Allopurinol"[Mesh]) OR ("Atovaquone"[Mesh] OR "atovaquone, proguanil drug combination" [Supplementary Concept]))) OR ((pentamidine[Title/Abstract] OR ambisome[Title/Abstract] OR amphotericin[Title/Abstract] OR paromomycin[Title/Abstract] OR miltefosine[Title/Abstract] OR pentavalent[Title/Abstract] OR sodium[Title/Abstract] OR sitamaquine[Title/Abstract] OR azole*[Title/Abstract] OR allopurinol[Title/Abstract] OR atovaquone[Title/Abstract] OR ketoconazole[Title/Abstract] OR fluconazole[Title/Abstract] OR metronidazole[Title/Abstract])))) AND (((("Leishmaniasis, Visceral"[Mesh]) OR visceral leishmaniasis[Title/Abstract]) OR kala azar[Title/Abstract]) OR black fever[Title/Abstract])))) NOT (((animals not humans)))
Filters activated: Publication date from 2016/01/01 to 2020/12/31.

- This search strategy uses the PubMed RCT filter for sensitivity- and precision-maximising version (2008). https://work.cochrane.org/pubmed

Database: Embase 1974 to present

Search Strategy:

----

1 exp visceral leishmaniasis/ (9675)
2 "black fever".ti,ab. (17)
3 "kala azar".ti,ab. (2227)
4 "visceral leishmaniasis".ti,ab. (9360)
5 1 or 2 or 3 or 4 (12383)
6 exp randomized controlled trial/ (603493)
7 Controlled clinical study/ (464274)
8 Random$.ti,ab. (1531396)
9 randomization/ (86779)
10 intermethod comparison/ (260073)
11 placebo.ti,ab. (305154)
12 (compare or compared or comparison).ti. (507638)
13 ((evaluated or evaluate or evaluating or assessed or assess) and (compare or compared or comparing or comparison)).ab. (2100417)
14 (open adj label).ti,ab. (78868)
15 ((double or single or doubly or singly) adj (blind or blinded or blindly)).ti,ab. (231216)
16 double blind procedure/ (172220)
17 parallel group$1.ti,ab. (25399)
18 (crossover or cross over).ti,ab. (104779)

For peer review only

19 ((assign$ or match or matched or allocation) adj5 (alternate or group$1 or intervention$1 or patient$1 or subject$1 or participant$1)).ti,ab. (328145)
20 (assigned or allocated).ti,ab. (386517)
21 (controlled adj7 (study or design or trial)).ti,ab. (346624)
22 (volunteer or volunteers).ti,ab. (245818)
23 trial.ti. (298535)
24 6 or 7 or 8 or 9 or 10 or 11 or 12 or 13 or 14 or 15 or 16 or 17 or 18 or 19 or 20 or 21 or 22 or 23 (4650673)
25 5 and 24 (1506)
26 (pentamidine or ambisome or amphotericin or paromomycin or miltefosine or pentavalent or sodium or sitamaquine or azole* or allopurinol or atovaquone or ketoconazole or fluconazole or metronidazole).mp. (1012481)
27 5 and 26 (3701)
28 25 or 27 (4786)
29 (exp animal/ or animal.hw. or nonhuman/) not (exp human/ or human cell/ or (human or humans).ti.) (6396469)
30 28 not 29 (3730)
31 30 (3730)
32 limit 31 to yr="2016 -Current" (774)

- This search strategy uses the Cochrane RCT filter for Embase. https://www.cochranelibrary.com/central/central-creation

Scopus

((TITLE-ABS-KEY ("visceral leishmaniasis" OR "kala azar" OR "black fever")) AND ((TITLE-ABS-KEY (random* OR rct OR placebo OR allocat* OR crossover* OR "cross over" OR trial OR (doubl* W/1 blind*) OR (singl* W/1 blind*))) OR (TITLE-ABS-KEY (control* W/1 trial*)) OR (TITLE-ABS-KEY (cohort*)))) OR ((TITLE-ABS-KEY ("visceral leishmaniasis" OR "kala azar" OR "black fever")) AND (TITLE-ABS-KEY (pentamidine OR ambisome OR amphotericin OR paromomycin OR miltefosine OR pentavalent OR sodium OR sitamaquine OR azole* OR allopurinol OR atovaquone OR ketoconazole OR fluconazole OR metronidazole))) AND (EXCLUDE (EXACTKEYWORD , "Animals") OR EXCLUDE (EXACTKEYWORD , "Animal") OR EXCLUDE (EXACTKEYWORD , "Animal Experiment") OR EXCLUDE (EXACTKEYWORD , "Mouse") OR EXCLUDE (EXACTKEYWORD , "Mice") OR EXCLUDE (EXACTKEYWORD , "Dogs")) AND (LIMIT-TO (LIMIT-TO (PUBYEAR , 2020) OR (PUBYEAR , 2019) OR LIMIT-TO (PUBYEAR , 2018) OR LIMIT-TO (PUBYEAR , 2017) OR LIMIT-TO (PUBYEAR , 2016))

Web of Science Core Collection

1. TOPIC: ("visceral leishmaniasis" OR "kala azar" OR "black fever")
2. TOPIC: (random* OR rct OR placebo OR allocat* OR crossover* OR "cross over" OR trial OR (doubl* near/1 blind*) OR (singl* near/1 blind*))
3. TOPIC: (control* near/1 trial*)
4. TOPIC: (cohort*)
5. #4 OR #3 OR #2
6. #5 AND #1
7. TOPIC: (pentamidine OR ambisome OR amphotericin OR paromomycin OR miltefosine OR pentavalent OR sodium OR sitamaquine OR azole* OR allopurinol OR atovaquone OR ketoconazole OR fluconazole OR metronidazole)

For peer review only

8. #7 AND #1
9. #8 OR #6
10. #8 OR #6
11. Refined by: PUBLICATION YEARS: (2020 OR 2019 OR 2018 OR 2017 OR 2016)

Cochrane Central Register of Controlled Trials
Issue 5 of 12, May 2020

#1	MeSH descriptor: [Leishmaniasis, Visceral] explode all trees	39
#2	"black fever"	1
#3	"kala azar"	119
#4	"visceral leishmaniasis"	242
#5	#1 or #2 or #3 or #4	283

Custom Range: 2016 to 2020

World Health Organization Global Index Medicus https://www.globalindexmedicus.net/

tw:(tw:(("visceral leishmaniasis" OR "kala azar" OR "black fever")) AND (instance:"ghl") AND (year_cluster:("2016" OR "2017" OR "2018" OR "2019" OR “2020”))) AND (instance:"ghl")

ClinicalTrials.gov Advanced Search https://clinicaltrials.gov/ct2/search/advanced?

Condition or disease: visceral leishmaniasis OR kala azar OR black fever

WHO International Clinical Trials Registry Platform http://apps.who.int/trialsearch/

visceral leishmaniasis OR kala azar OR black fever – Trials at ALL stages
2016 – date

NOTE: THIS PLATFORM IS UNAVAILABLE ON 14/05/2020

For peer review only

PRISMA-P (preferred reporting items for systematic review and meta-analysis protocols) 2015 checklist: recommended items to address in a systematic review protocol

Section and topic	Item No	Checklist item
**Administrative information**<br>Authors: All page numbers relates to the clear revised submission version of the manuscript submitted On the 10th Oct 2023.		
Title:		
Identification	1a	A protocol for individual participant data meta-analysis; page 1; line 2-3.
Update	1b	Not applicable
Registration	2	PROSPERO registration: CRD42021284622; page 4; lines 72-73.
Authors:		
Contact	3a	Name with institutional affiliations of all authors and e-mail address corresponding authors; pages 1-2; lines 9-39.<br>E-mail address of all authors; page 2; lines 22-39
Contributions	3b	Authors' contribution; page 21; line 388-394.
Amendments	4	Not applicable
Support:		
Sources	5a	Funding; page 21; line 395-398.
Sponsor	5b	Funding; page 21; line 395-398.
Role of sponsor or funder	5c	Funding; page 21; line 395-398.
**Introduction**		
Rationale	6	Introduction; page 6; lines 95-112.
Objectives	7	Study objectives; pages 6-7; lines 113-123.
**Methods**		
Eligibility criteria	8	Elements of PICOT & study and patient eligibility criteria:<br>Pages 7-8; Lines 124-155
Information sources	9	Identification of relevant studies using IDDO VL library<br>Pages 9-10: Lines 176-186
Search strategy	10	Identification of relevant studies using IDDO VL library

Section and topic	Item No	Checklist item
		Pages 9-10: Lines 176-186
Study records:		
Data management	11a	Collating IPD & Data management; page 10; lines 187-200.
Selection process	11b	Identification of relevant studies using IDDO VL library<br>Pages 9-10: Lines 176-186
Data collection process	11c	Collating IPD & Data management; page 10; lines 187-200.
Data items	12	Statistical methods for primary and secondary outcomes; page 11-12; lines 201-261
Outcomes and prioritization	13	Outcomes and definitions;<br>page 8-9; lines 157-173.
Risk of bias in individual studies	14	Risk of bias assessment in included studies<br>page 16; lines 318-328
Data synthesis	15a	Handling missing data; page 14; lines 27-276.
	15b	Assessment of risk of potential bias in missing studies; pages 16-17; line 329-335.
	15c	Subgroup analyses: Page 16; lines 308-317<br><br>Sensitivity analyses; page 14; lines 277-285.
	15d	Assessment of risk of potential bias in missing studies; pages 16-17; line 329-335.
Meta-bias(es)	16	Risk of bias assessment in included studies<br>page 16; lines 318-328
Confidence in cumulative evidence	17	Risk of bias assessment in included studies<br>page 16; lines 318-328
